# Isolation and Identification of Antitrypanosomal and Antimycobacterial Active Steroids from the Sponge *Haliclona simulans*

**DOI:** 10.3390/md12052937

**Published:** 2014-05-16

**Authors:** Christina Viegelmann, Jennifer Parker, Thengtheng Ooi, Carol Clements, Gráinne Abbott, Louise Young, Jonathan Kennedy, Alan D. W. Dobson, RuAngelie Edrada-Ebel

**Affiliations:** 1 Strathclyde Institute of Pharmacy and Biomedical Sciences, University of Strathclyde, The John Arbuthnott Building, 161 Cathedral Street, Glasgow, Scotland G4 0RE, UK; E-Mails: jennifer.parker.100@strath.ac.uk (J.P.); thengthengooi@hotmail.com (T.O.); thengthengooi@hotmail.com (C.C.); thengthengooi@hotmail.com (G.A.); louise.c.young@strath.ac.uk (L.Y.); 2 Marine Biotechnology Centre, Environmental Research Institute, University College Cork, Lee Road, Cork, Ireland; E-Mails: jonathan.kennedy@ucc.ie (J.K.); A.Dobson@ucc.ie (A.D.W.D.)

**Keywords:** *Haliclona simulans*, marine sponges, steroids, antitrypanosomal, anti-mycobacterial

## Abstract

The marine sponge *Haliclona simulans* collected from the Irish Sea yielded two new steroids: 24-vinyl-cholest-9-ene-3β,24-diol and 20-methyl-pregn-6-en-3β-ol,5α,8α-epidioxy, along with the widely distributed 24-methylenecholesterol. One of the steroids possesses an unusually short hydrocarbon side chain. The structures were elucidated using nuclear magnetic resonance spectroscopy and confirmed using electron impact- and high resolution electrospray-mass spectrometry. All three steroids possess antitrypanosomal and anti-mycobacterial activity. All the steroids were found to possess low cytotoxicity against Hs27 which was above their detected antitrypanosomal potent concentrations.

## 1. Introduction

Since the discovery of penicillin and its widespread use during World War II, many people have become complacent about infectious diseases, believing that with antibiotics, death due to infection has become a thing of the past. However, in 2004, infectious and parasitic diseases ranked as the second leading cause of death in the world, after cardiovascular diseases, with respiratory infections considered as the fourth and fifth most common cause of death in women and men respectively, worldwide [1].

Trypanosomiasis is a vector-transmitted disease caused by parasitic protozoa called trypanosomes. Both American trypanosomiasis (Chagas disease) and human African trypanosomiasis (sleeping sickness) are included in the list of the World Health Organization’s neglected tropical diseases [2]. Chagas disease, caused by *Trypanosoma cruzi*, infects an estimated 7–8 million people worldwide, although infection predominantly occurs in Latin America and spreads to other countries due to travel by infected persons. Sleeping sickness affects an estimated 20,000 people a year. Approximately 98% of reported cases are the chronic form of sleeping sickness caused by infection with *T. brucei gambiense* whereas the acute form, due to infection with *T. b. rhodesiense*, accounts for 2% of reported cases [2]. Currently, only a few medicines are registered for trypanosomiasis and many of them have serious adverse effects [3,4]. In addition, both Chagas disease and sleeping sickness are expensive to treat. As such, one of the targets of the WHO is to provide cheaper, more effective, and less toxic medicines for the management and elimination of these diseases [2].

Tuberculosis (TB) is a disease caused by *Mycobacterium tuberculosis*, a Gram-positive, acid-fast bacillus. The most common form of tuberculosis is pulmonary; however, extrapulmonary TB can also occur. TB, if left untreated, is an often-fatal disease with up to 70% of HIV negative patients dying within 10 years. The mortality rate is higher for HIV positive patients with untreated TB as the average survival rate becomes less than 6 months [5]. Although treatment is available, TB remains the second leading cause of death from infectious disease in the world, after HIV [6]. Despite the consistent decrease in incidence and mortality of TB cases globally since 2006 and probability that the Millennium Development Goal of 2015 will be achieved, the global burden of TB is still high. Approximately 8.7 million new cases of TB and 1.4 million deaths due to TB were reported in 2011 [6]. In addition, there is insufficient data to determine the global trend of multidrug-resistant TB (MDR-TB), largely due to lack of testing for resistance in many countries. However, in 2011 the estimated number of MDR-TB cases was 310,000. At least one case of extensively drug-resistant TB (XDR-TB) has been reported in 84 countries [6].

It has been approximately half a century since the first-line TB drugs were first used. New medicines are necessary in order to combat MDR-TB and XDR-TB, shorten the current treatment regimens, treat latent TB in patients that are infected with the bacteria but have not developed the disease, and to improve the prognosis of patients with both TB and HIV. Eleven prospective TB drugs are undergoing clinical trials. Research is also underway on TB vaccines, with 12 in clinical trials [6]. While this is encouraging, it is still important to continue the search for lead compounds with different mechanisms of action.

Sterols have shown promise as new antitrypanosomal and anti-mycobacterial compounds. Recent research has shown that steroids may be used to treat trypanosomiasis by inhibiting the glucose-6-phosphate dehydrogenase (G6PD) enzyme in trypanosomes [7,8] which is the enzyme involved in the first committed step of the pentose-phosphate pathway. This prevents the formation of NADPH, increasing the susceptibility of the trypanosomes to oxidative stress [8]. The sterol biosynthetic pathway of trypanosomes has also come into focus. 24-Sterol methyltransferase (24-SMT) and sterol 14α-demethylase (CYP51) are two examples of enzymes that serve as targets for new antitrypanosomal drugs [9,10,11]. The genus *Mycobacterium* has also been reported to possess a sterol biosynthetic pathway that is homologous to that of the yeast, *Saccharomyces cerevisiae* [12]. Sterol 14α-demethylase is also a target in *M. tuberculosis* [13]. Sterols could therefore prove to be interesting lead compounds for the treatment of *Mycobacterium* sp. infections.

Sponges (Phylum Porifera) are primitive metazoic organisms that are among the oldest multicellular animals in the world [14]. Despite their simple morphology, marine sponges are well-known for being abundant sources of novel compounds, particularly steroids with a diverse range of conventional and unconventional side chains and nuclei [15,16,17]. Interest in sponge metabolites began in the 1950s when Bergmann and Feeney first reported the isolation of two sponge nucleosides, spongouridine and spongothymidine [18,19,20]. The knowledge gained from this discovery eventually led to the development of several FDA- and EMEA-approved drugs: cytarabine arabinoside (Ara-C), an anti-leukemic agent, adenine arabinoside (Vidarabine or Ara-A), an antiviral agent, and even azidothymidine (AZT) which is used in the treatment of HIV [21].

The sponge *Haliclona simulans* belongs to the family *Chalinidae* of the order *Haplosclerida* and the class *Demospongiae* [22]. The genus *Haliclona* has already given rise to many interesting metabolites, with as many as 190 compounds of various chemical classes and functions having been reported [23]. *Haliclona simulans*, by virtue of its genus, which has already produced a large quantity of active metabolites, therefore appears to be a very promising source of lead compounds. This paper describes the isolation and identification of three antitrypanosomal and anti-mycobacterial steroids from the Irish Sea sponge *Haliclona simulans*.

## 2. Results and Discussion

**Figure 1:**
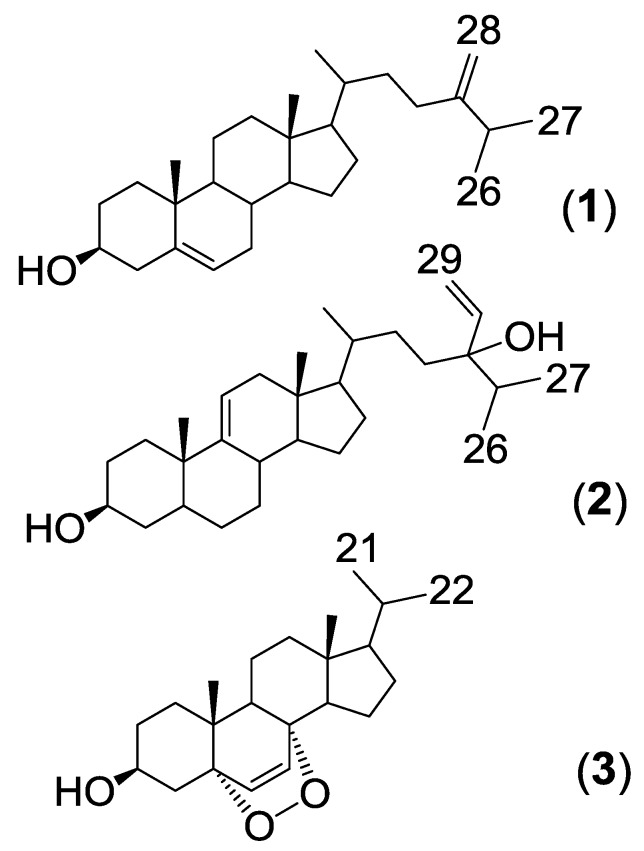
Structures of steroids isolated from *H. simulans*.

Three steroids (Figure 1) were isolated from *Haliclona simulans* following HP20 chromatography and flash chromatography. One steroid was identified as 24-methylenecholesterol (**1**) whereas the other two were unidentified based on a literature search. These steroids were found to be active against *Trypanosoma brucei brucei* and *Mycobacterium marinum*.

24-Methylenecholesterol (**1**) was the major non-polar compound present in the sponge. It was first isolated from the sponge *Chalina arbuscula* Verill [24] and has since been isolated from a variety of marine sources, including other *Haliclona* sponges [17]. 24-Methylenecholesterol has been reported as a precursor to many other sponge sterols [25]. The ^1^H and ^13^C NMR data (Table 1 and Table 2) of the 24-methylenecholesterol isolated in this study closely matched those reported in the literature [26]. The orientation of the 3-hydroxyl group was determined using ROESY and by analysis of the coupling constants. The large *J* value of H-3 (10.98 Hz) indicated axial-axial correlations. H-3 was therefore assigned an α-orientation whereas OH-3 was designated a β-orientation. The steroid was derivatised using MSTFA to facilitate ionization using GCMS. EI-GCMS showed a peak with *m/z* 470.4 [M]^+^ which corresponded to a TMS derivatized 24-methylenecholesterol.

**Table 1:** ^1^H (400 MHz) NMR chemical shifts (CDCl_3_) of the steroids isolated from *H. simulans*.

Position	1	2	3
1	1.81 (d, 5.1)	1.82 (d, 11.5)	1.68, 1.94 (d, 4.6)
2	1.50	1.45 (d, 12.9)	1.52, 1.83
3	3.52 (td, 5.3, 11.0)	3.51 (tt, 4.7, 10.6)	3.96 (tt, 5.1, 11.1)
4	2.27 (m)	2.24 (m)	1.90 (s), 2.12 (d, 4.8)
6	5.34 (d, 5.7)	0.90 (d, 4.0)	6.23 (d, 8.5)
7	1.95		6.49 (d, 8.5)
9			1.50 (m)
11		5.32 (d, 5.0)	1.20, 1.49
12		1.14, 1.94	1.21, 1.97
14		1.06	1.57, (d, 3.6)
15			0.99
16			1.24 (s)
17			1.20
18	0.67 (t, 3.1)	0.66 (s)	0.80 (s)
19	0.99 (s)	0.98 (s)	0.87 (s)
20		1.41 (d, 3.9)	1.98 (m)
21	0.93 (d, 6.7)	0.93 (d, 6.3)	0.98 (d, 7.0)
22		1.85	1.02 (d, 7.0)
23		1.11, 1.99	
25		1.99	
26	1.02 (d, 2.3)	0.98	
27	1.02 (d, 2.3)	0.87	
28	4.64 (s), 4.70 (s)	5.72 (m)	
29		5.12 (d, 17.9) 5.25 (d, 11.4)	

**Table 2:** ^13^C (100 MHz) NMR chemical shifts (CDCl_3_) of the steroids isolated from *H. simulans*.

Position	1	2	3
1	37.3 (CH_2_)	37.3 (CH_2_)	34.8 (CH_2_)
2	31.7 (CH_2_)	31.6 (CH_2_)	30.2 (CH_2_)
3	71.9 (CH)	71.9 (CH)	66.6 (CH)
4	42.3 (CH_2_)	42.3 (CH_2_)	37.0 (CH_2_)
5	140.8 (C)	50.2 (CH)	82.2 (C)
6	121.8 (CH)	34.8 (CH_2_)	135.50 (CH)
7	31.0 (CH_2_)	22.0 (CH_2_)	130.8 (CH)
8	32.0 (CH)	21.1 (CH)	79.5 (C)
9	50.2 (CH)	140.8 (C)	51.1 (CH)
10	36.6 (C)	36.6 (C)	36.0 (C)
11	21.2 (CH_2_)	121.8 (CH)	23.5 (CH_2_)
12	39.8 (CH_2_)	39.8 (CH_2_)	39.5 (CH_2_)
13	42.4 (C)	42.4 (C)	44.8 (C)
14	56.8 (CH)	56.0 (CH)	51.7 (CH)
15	24.4 (CH_2_)	24.4 (CH_2_)	20.7 (CH_2_)
16	28.3 (CH_2_)	29.8 (CH_2_)	29.8 (CH_2_)
17	56.1 (CH)	56.8 (CH)	56.2 (CH)
18	11.9 (CH_3_)	11.9 (CH_3_)	12.7 (CH_3_)
19	19.5 (CH_3_)	19.5 (CH_3_)	18.3 (CH_3_)
20	35.9 (CH_2_)	36.3 (CH)	33.9 (CH)
21	18.8 (CH_2_)	19.0(CH_3_)	22.1 (CH_3_)
22	23.9 (CH_2_)	28.3 (CH_2_)	28.4 (CH_2_)
23	34.8 (CH_2_)	28.5 (CH_2_)	28.1 (CH_3_)
24	157.0 (C)	89.2 (C)	
25	33.9 (CH)	32.0 (CH)	
26	22.1 (CH_3_)	17.8 (CH_3_)	
27	28.1 (CH_3_)	16.7 (CH_3_)	
28	106.0 (CH_2_)	137.2 (CH)	
29		116.3 (CH_2_)	

**Figure 2:**
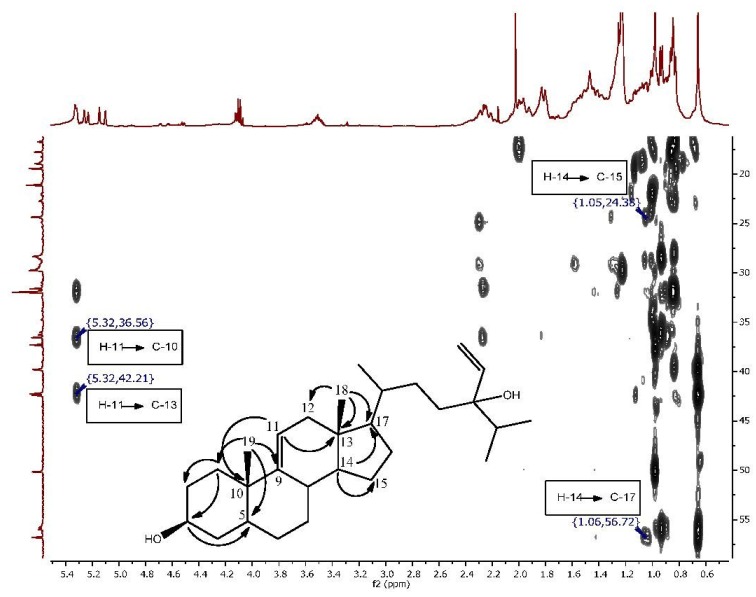
Expansion of the HMBC spectrum highlighting the correlations which indicate the change in the position of the double bond in Compound **2**.

The second isolated steroid (**2**) is a derivative of saringosterol, which was first isolated from a brown algae [27]. The difference lies in the position of the double bond of the steroid nucleus. In saringosterol it is found in the Δ^5(6)^ position, whereas in this derivative from *H. simulans* it is found in the Δ^9(11)^ position. The ^1^H and ^13^C NMR data (Table 1 and Table 2, Supplementary Figures S1 and S3–S5) of the saringosterol derivative were comparable to the values found in the literature [28]. The olefinic system of the steroid nucleus was elucidated using the COSY (Supplementary Figures S2 and S8) and HMBC spectra as shown in Figure 2, Supplementary Figures S6, S8, and S9. The broad doublet at δ_H_ 5.32 assigned to H-11 showed the coupling of the olefinic hydrogen with one of the two protons at H-12 (δ_H_ 1.94). The HMBC confirmed the position of C-12 as it is one of the three carbons correlating with the methyl group on C-13. Crosspeaks were observed from C*H*_3_-18 (δ_H_ 0.66) to C-12 (δ_C_ 39.8), C-13 (δ_C_ 42.4) and C-17 (δ_C_ 56.8). HMBC correlations of H-14 (δ_H_ 1.06) with C-15 (δ_C_ 24.4), and of H-14 to C-17 were also visible. The doublet for H-11 at δ_H_ 5.32 correlated with C-10 (δ_C_ 36.6) and C-13 (δ_C_ 42.4) (Supplementary Figure S7). The β-orientation of 3-OH was once again determined using the ROESY spectrum and the *J* values (tt, 4.7, 10.6 Hz) of the H-3 peak at 3.51 ppm. High resolution ESI-MS analysis of compound **2** showed an ion peak at *m/z* 429.37303 [M + H]^+^ which corresponded to the molecular formula of C_29_H_49_O_2 _(Supplementary Figure S11). Sterol **2** was elucidated as 24-vinyl-cholest-9-ene-3β,24-diol (Figure 1 and Supplementary Figure S10).

Another new sterol (**3**) was also isolated (Figure 1, Supplementary Figures S12, S14–S16; Table 1 and Table 2). It eluted later from the generic flash silica column than the previous two sterols as it is more polar. This sterol possessed a 5α,8α-epidioxy nucleus with a shortened side chain. The two doublets at δ_H_ 6.23 and 6.49 (H-6 and H-7, respectively) were shown on the COSY (Supplementary Figure S13) to form an isolated spin system. The HMBC spectrum confirmed that H-6 and H-7 correlated with oxygenated carbons at δ_C_ 82.2 and 79.5 (C-5 and C-8 respectively). The orientation of the 5,8-epidioxy peroxide group was confirmed by comparing the chemical shifts and coupling patterns of H-6 and H-7 (Supplementary Figure S12) to the values found in literature. A 5α,8α-epidioxy group results in chemical shifts of 6.5 and 6.3 ppm for H-6 and H-7 whereas a 5β,8β-epidioxy group would result in chemical shifts of 5.9 and 5.6 respectively [29].

**Figure 3:**
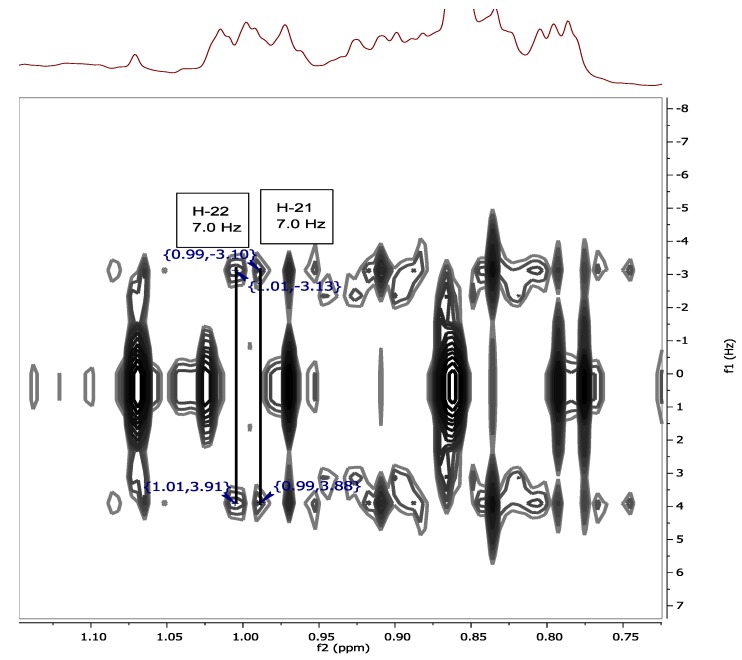
*J*-resolved spectrum showing the splitting of H-21 (δ_H_ 0.98) and H-22 (δ_H_ 1.02).

**Figure 4:**
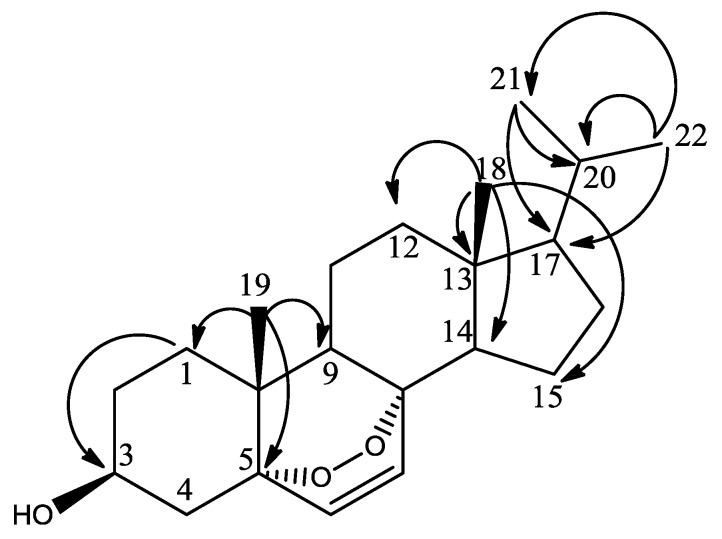
HMBC correlations (H to C) of methyl groups in sterol **3**.

The chemical shifts of the methyl groups on C-20 were assigned based on the COSY (Supplementary Figure S13) and *J*-resolved spectra (Figure 3, Supplementary Figures S18 and S19). The *J*-resolved spectra showed that the peaks at δ_H_ 0.98 (H-21) and δ_H_ 1.02 (H-22) were doublets; therefore, both methyl groups must be attached to C-20. The COSY spectrum showed that both C*H_3_*-21 and C*H_3_*-22 correlated with a peak at δ_H_ 1.98, which was assigned to H-20. C*H_3_*-21 and C*H_3_*-22 were also correlating on the HMBC with C-17 as shown in Figure 4, Supplementary Figures S17 and S20. Sterol **3** gave a retention time of 23.67 min on a C_18_-HPLC column and exhibited an ion peak at *m/z* of 347.2580 [M + H]^+^ by high resolution ESI-MS with a molecular formula of C_22_H_35_O_3_ (Supplementary Figure S22). The structure of sterol **3** was elucidated as 20-methyl-pregn-6-en-3β-ol, 5α,8α-epidioxy (Supplementary Figure S21).

**Figure 5:**
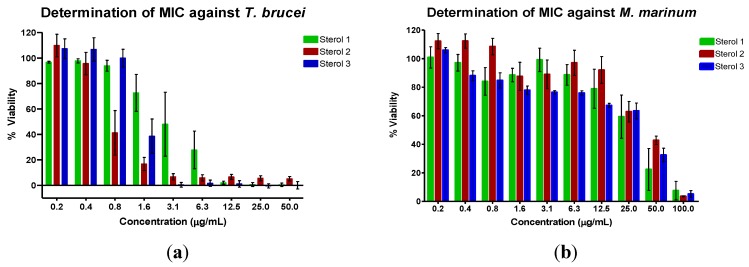
Determination of the minimum inhibitory concentration value (MIC) of the sterols against (**a**) *T. b. brucei* and (**b**) *M. marinum*. The assay was performed using various concentrations of the sterols in μg/mL. The mean values were then converted to μM.

The inhibitory activities of the three sterols against *Trypanosoma brucei brucei* and *Mycobacterium marinum* are shown in Table 3 and Figure 5. Suramin, the positive control for the *T. brucei* assay, is the treatment of choice for stage 1 sleeping sickness [3]. The positive control for the *M. marinum* assay was gentamicin, an aminoglycoside antibiotic. Sterol **1**, 24-methylenecholesterol, was the most potent against *M. marinum* but the least active against *T. b. brucei*. The MIC against *M. marinum* was less than that of 24-methylenecholesterol isolated from the sponge *Svenzea zeai* against *M. tuberculosis* (MIC: 120.1 μg/mL or 251.26 μM) [30]*.* Sterol **2**, the saringosterol derivative, was the most active against *T. b. brucei*. Saringosterol has been reported to have an IC_50_ of 7.8 ± 1.2 μM against *T. b. brucei* [31]. The shift in double bond position from Δ^5(6)^ to Δ^9(11)^ in the new congener seems to improve the antitrypanosomal activity of saringosterol. Steroids with the Δ^9(11)^ double bond have previously been isolated from marine invertebrates such as starfish [32,33,34,35] and sea cucumbers [36,37], as well as from another sponge, *Haliclona rubens* (renamed as *Amphimedon compressa*) [38]. The activity of saringosterol against *M. tuberculosis* has also been previously reported (MIC: 0.25 μg/mL or 0.58 μM) [39]. The change in the position of the double bond may therefore have had a detrimental effect on the anti-mycobacterial activity of the steroid. Saringosterol is often isolated as a mixture of 24*R*/*S* epimers [31,39] although the 24*R* epimer has been found to be more active than the 24*S* epimer against *M. tuberculosis* [39]. The optical rotation of sterol **2** in this study was determined to be −4.5, indicating that it is likely a mixture of epimers which may plausibly explain the loss of activity in the isolated congener. In addition, the test microorganisms that were used were different, although *M. marinum* is often used as the model organism for *M. tuberculosis*.

**Table 3:** Antimicrobial activities and cytotoxicity on Hs27 cells of *H. simulans* sterols.

Sterol	MIC Average ± Std Dev (*n* = 4)		Cytotoxicity on HS27 Cells
	*T. b. brucei* (μM)	*M. marinum* (μM)	IC_50_ (μM) ± Std Dev (*n* = 3)
**1**	21.56 ± 11.80	156.90 ± 54.35	58 ± 3.53
**2**	4.58 ± 1.80	233.44 ± 0	>100 ± 4.03
**3**	9.01 ± 0	288.81 ± 0	100 ± 2.95
**Suramin**	0.11 ± 0		
**Gentamycin**		13.48 ± 0	

Sterol **3** was the next most active against *T. b. brucei*.The 5α,8α-epidioxy sterol could be an artefact, a result of oxidation of a ∆^5,7^-sterol, as has been observed in *Dysidea* sp. [40]. However, 5α,8α-epidioxy sterols have previously been isolated from the sponge *Luffariella* cf. *variabilis* and these sterols are believed to be natural products and not artifacts as the isolation was performed rapidly and no precursors were found [29]. In addition, some fungi produce these epidioxy sterols, such as ergosterol peroxide [41], and so it cannot be ruled out that **3** is a natural product of *H. simulans* or even of an endosymbiont. The peroxide functional group may contribute to the antitrypanosomal activity. 24-hydroperoxy-24-vinylcholesterol, a peroxide derivative of saringosterol, was reported to have an IC_50_ of 3.2 M against *T. b. brucei* as opposed to that of saringosterol which was 7.8 μM [31]. Ergosterol peroxide, a fungal metabolite, has also been known to have antitrypanosomal activity, although the assay was performed against *T. cruzi* [42]. It is also active against *M. tuberculosis* (MIC 3.5 μM) and indeed was the most active of the nine steroids that were assayed in the study [43]. Another endoperoxide-containing natural product well-known for its anti-protozoal activity is artemisinin. Short side chain sterols are uncommon but have previously been isolated from marine invertebrates, including from tunicates, gorgonians and sponges [44,45]. These sterols may be *in vivo* oxidative products from sterols with unsaturated side chains and not degradation products obtained during laboratory work-up. A sterol bearing the same side chain as in **3** has been isolated from the gorgonian *Murecia californica* and the sponge *Damiriana hawaiiana* [45].

Previous studies have drawn some conclusions regarding the structure-activity relationships of sterols and their antitrypanosomal and anti-mycobacterium activity. Hydroxylated side chains, such as the one proposed for sterol **2**, result in an increase in antitrypanosomal activity [31]. The peroxide functional group on sterol **3** may also contribute to activity, as 24-hydroperoxy-24-vinylcholesterol, a peroxide derivative of saringosterol, had an IC_50_ of 3.2 μM against *T. b. brucei* whereas saringosterol had an IC_50_ of 7.8 μM against the same microorganism [31]. Another functional group of sterols that contributes to bioactivity is the 3β-hydroxy group, which is required for the inhibition of 24-sterol methyltransferase of *Leishmania major* by azasterols [46] as well as for the binding of sterols to plant yeast SMT [47]. As previously mentioned, 24-SMT is also a target for antitrypanosomal drugs. Although the mechanism of action of the sterols in this study is unknown, it is possible that they act by inhibiting 24-SMT and thereby disrupting the sterol biosynthetic pathway in *T. b. brucei*. The 3-hydroxy group, as well as a 14α-methyl group, is also essential for the binding of sterols to sterol 14α-demethylase in *M. tuberculosis* [13]. Although the three sterols isolated from *H. simulans* all contain the 3β-hydroxy moiety, none possess the 14α-methyl group required to bind to this enzyme. It is possible that they act upon another enzyme in the pathway or by a different mechanism altogether. The steroid nucleus itself also affects the anti-mycobacterium activity of the compound, as it has been found that a 5(6→7)abeo-steroidal nucleus leads to an increase in the potency of the compound [30]. The effect of the presence of the double bond at Δ^9(11)^ on the antimicrobial activity of steroids has not been studied; however, Δ^9(11)^ steroids have found applications as lead compounds for anti-inflammatory drugs as they possess fewer side effects than the conventional corticosteroids [48]. It would therefore be of interest to determine the advantages of Δ^9(11)^ steroids as antimicrobial agents.

**Figure 6:**
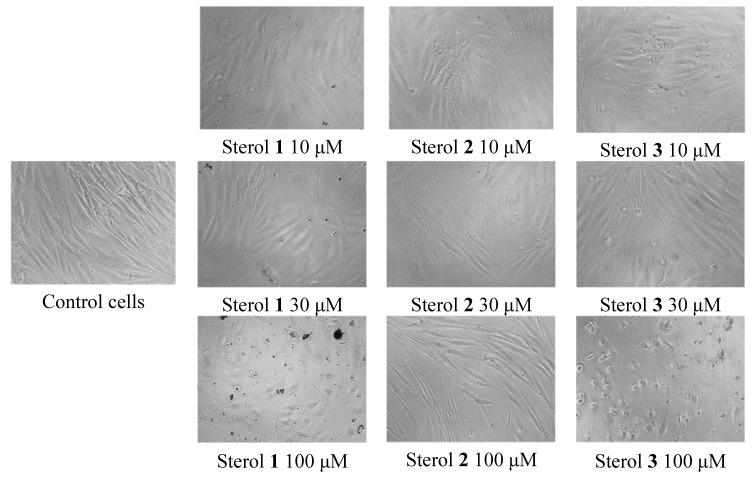
Change in morphology of normal fibroblasts Hs27 observed under the microscope in the cell cytotoxicity assay of *H. simulans* sterols.

All sterols at concentration ranges of 0.1 to100 μM were subjected to cell cytotoxicity assay on normal fibroblasts derived from human foreskin (Hs27 cells). The most antitrypanosomal active saringosterol congener (**2)** showed no cytoxicity and no difference with the control in terms of changes in cell morphology of Hs27 (Figure 6). Sterols **1** and **3** exhibited cytotoxicity at 58 and 100 μM, respectively as shown in Table 3. At 100 μM concentration of sterols **1** and **3**, the Hs27 shrunk 10× to irregularly shaped cells (see Figure 6). The cytoxicity of sterols **1** and **3** is still above their dosage threshold as potential antitrypanosomal drugs.

## 3. Experimental Section

### 3.1. Acquisition of Sponge Sample

The sponge, *Haliclona simulans*, was collected from Kilkieran Bay, Galway, Ireland by the Environmental Research Institute, University College Cork. It was freeze-dried, sealed under vacuum and sent to the Strathclyde Institute of Pharmacy and Biomedical Sciences, University of Strathclyde where it was stored at −20 °C until the extraction of its metabolites.

### 3.2. Extraction and Isolation of Sponge Metabolites

The freeze-dried sponge (dry weight: 107.20 g) was finely ground using an analytical mill. The powder was macerated in acetone at room temperature with constant stirring for three hours. This was repeated three times, after which the extraction procedure was performed with methanol. The acetone and methanol extracts were combined and subjected to Diaion^®^ HP20 (Mitsubishi Chemical Corporation, Tokyo, Japan) chromatography. The compounds were eluted using a stepwise gradient of 100% water to 100% methanol in 10% increments. The column was washed with 50:50 acetone methanol and 100% methanol. These non-polar wash fractions were pooled and further fractionated using the Reveleris^®^ Flash Forward system (Grace Davison Discovery Sciences, Columbia, MD, USA). The sample was loaded onto a generic silica 24 g/32 mL (20 × 130 mm) column and eluted in a three-step gradient (5 min: 100% hexane, 45 min: 100% hexane to 100% ethyl acetate, 10 min: 100% ethyl acetate) at a flow rate of 10 mL/min. The sterols eluted at approximately 26–30 min (**1**), 31–32 min (**2**) and 35–36 min (**3**) with following yield 334.8 mg = 0.31%, 11.5 mg = 0.011%, 6.7 mg = 0.006%, respectively.

24-Methylenecholesterol (**1)**. White, needle-like crystals; [α]_D_^20^ −23 (*c* 0.1, CHCl_3_); *R*_f_: 0.52 (TLC silica, 95:5 CH_2_Cl_2_); EIMS: *m/z* 470.4 [M + CH_3_Si]^+^ (derivatised steroid), calculated for C_31_H_54_OSi, 470.3938.

24-Vinyl-cholest-9-ene-3β,24-diol (**2**). Very fine crystals; [α]_D_^20^ −4.5 (*c* 0.1, CHCl_3_); *R*_f_: 0.42 (TLC silica, 95:5 CH_2_Cl_2_); HRESIMS *m/z* 429.3731 [M + H]^+^, calculated for C_29_H_49_O_2_, 429.3727.

20-Methyl-pregn-6-en-3β-ol, 5α,8α-eipidioxy (**3**). Small crystals; [α]_D_^20^ +5 (*c* 0.1, CHCl_3_); *R*_f_: 0.36 (TLC silica, 95:5 CH_2_Cl_2_:MeOH); HRESIMS *m/z* 347.2580 [M + H]^+^, calculated for C_22_H_35_O_3_, 347.2581.

### 3.3. Analysis of Sterols

NMR spectra were taken using a Jeol-LA400 FT-NMR spectrometer system (JEOL Ltd., Tokyo, Japan) with an AS400 magnet (Oxford Instruments, Inghilterra, UK) at 400 MHz for ^1^H and 100 MHz for ^13^C, using a Pulse Field Gradient “Autotune” 40TH5AT/FG broadband high sensitivity probe (JEOL Ltd., Tokyo, Japan ) to accept 5 mm tubes. LC-HRFTMS analysis was performed on a Dionex UltiMate-3000 (DIONEX, Sunnyvale, CA, USA) coupled to a ThermoScientific Exactive Orbitrap system (Thermo Fisher Scientific (Bremen) GmbH, Bremen, Germany). The column used was an ACE 5 C18 75 × 3.0 mm column from Hichrom Ltd., Reading, UK. Compounds were eluted with a flow rate of 300 μL/min using water (A) and acetonitrile (B), both of which contained 0.1% formic acid, by a gradient starting with 10% B and increasing to 100% B in 30 min. The mobile phase was maintained at 100% B for 5 min after which the column was equilibrated with 10% B. Sterol **2** eluted at 37.60 min and sterol **3** eluted at 23.67 min. The optical rotations of the compounds were measured using a Perkin-Elmer 341 polarimeter (Perkin-Elmer, Waltham,_MA, USA). The derivatisation of 24-methylenecholesterol was carried out by dissolving 1 mg of the steroid in 500 μL of acetonitrile. This was dried at 50 °C after which 200 μL of MSTFA (Sigma-Aldrich, St. Louis, MO, USA) was added and the sample heated at 80 °C for 30 min. The sample was then submitted for EI-GCMS using a Thermo Finnigan Polaris Q (Thermo Fisher Scientific (Bremen) GmbH, Bremen, Germany) with Trace GC. The column used was an Agilent DB5-ms UI (ID: 0.25 mm, length: 30 m, df: 0.25 μm, Agilent, Santa Clara, CA, USA).

### 3.4. Antitrypanosomal Assay

The sterols were prepared in stock solutions of 10 mg/mL in DMSO. Four microliters of these stock solutions were pipetted into one column of the transparent flat-bottomed 96-well plates after which 196 μL of HMI-9 was added, resulting in a concentration of 200 μg/mL. One-to-one serial dilutions with HMI-9 were carried out in the other columns of the plate. One hundred microliters of trypanosome suspension (containing *Trypanosoma brucei* S427 blood stream form at 3 × 10^4^ trypanosomes/mL) were added so that the final concentration of the compounds ranged from 100 μg/mL to 0.17 μg/mL. DMSO was included as a negative control at a concentration of 1% to 0.002% and suramin (Calbiochem-Novabiochem Co., La Jolla, CA, USA) was included as a positive control at a concentration range of 1 to 0.008 μM. The plate was incubated at 37 °C, 5% CO_2_ with a humidified atmosphere for 48 h, after which 20 μL of Alamar blue was added. The plate was incubated under the same conditions for another 24 h and the fluorescence read using the Wallac Victor microplate reader (Perkin Elmer, Cambridge, UK) with excitation at 530 nm and emission at 590 nm. The results were calculated as percentages of control values and the minimum inhibitory concentration values (MICs) were determined.

### 3.5. Anti-Mycobacterium Assay

*Mycobacterium marinum* ATCC.BAA535 from a thawed stock cryoculture was streaked onto Columbia (5% horse blood) agar slopes and incubated at 31 °C for 5 days. A loopful of the culture was then transferred into 10 mL of sterile 0.9% NaCl containing glass beads. The suspension was mixed and allowed to settle. Aliquots of the supernatant were added to a tube containing sterile 0.9% NaCl until the turbidity matched that of a 0.5 McFarland standard. A few drops of sterile 0.02% Tween 80 were added to homogenize the suspension. This was then shaken and the inoculum diluted 1 in 10 with cation-adjusted Mueller Hinton Broth (MHB) for use in the assay. The steroids were prepared in 10 mg/mL DMSO stock solutions and were diluted ten times to 1 mg/mL using cation-adjusted MHB. The final concentrations of the test solutions in the 96-well plate ranged from 100 to 0.17 μg/mL. DMSO was included as a negative control at a concentration range of 1% to 0.002% and gentamycin was included as a positive control at a concentration range of 100 to 0.78 μg/mL. One hundred microliters of the bacterial suspension was added to the wells. The plates were sealed and incubated at 31 °C for 5 days before the addition of 10 μL of Alamar blue. The plates were re-sealed and incubated at the same temperature for 24 h after which fluorescence was determined using the Wallac Victor microplate reader (excitation 530 nm, emission 590 nm). The results were calculated as percentages of control values and the minimum inhibitory concentration values (MICs) were determined.

### 3.6. Cell Cytotoxicity Assay

Normal fibroblasts derived from human foreskin (Hs27 cells*)* were obtained from ECACC (Sigma-Aldrich, Dorset, UK). They were cultured in DMEM media supplemented with 10% (v/v) foetal bovine serum, 2 mM glutamine and 50 μg/mL penicillin/streptomycin (all Invitrogen, Paisley, UK) solution in a humidified incubator at 37 °C in the presence of 5% CO_2_. Cells were routinely passaged at 90%–95% confluence.

Subsequently, cells were seeded at a concentration of 7500 cells/well in black 96 flat-bottomed plates and allowed to adhere overnight. After that time, Sterols **1**, **2** and **3** were added to the cells in the concentration range of 0.1 to100 μM (in half log units) and incubated for a further 48 h.

Viability was determined using a CellTiter-Glo^®^ (Promega, Southampton, UK) kit according to the manufacturer’s instructions. The resulting luminescence was measured using a Wallac Victor 2 (Perkin Elmer, Cambridge, UK). Results were expressed as “percentage of control” where the control is the luminescence value of cells in the absence of compound. All results were confirmed microscopically.

## 4. Conclusions

Sponges are recognized as possessing a diverse range of steroids that have both conventional and unconventional side chains and nuclei. This was confirmed by the diversity of nuclei and side chains isolated from *H. simulans*. Two novel sterols, 24-vinyl-cholest-9-ene-3β,24-diol and 20-methyl-pregn-6-en-3β-ol, 5α,8α-epidioxy were among the three obtained. The former sterol possessed a Δ^9(11)^ double bond that distinguished it from the known compound saringosterol, whereas the latter contained an endoperoxide moiety and an uncommonly short side chain. Both sterols were more active against *T. brucei* than the known sterol 24-methylenecholesterol, but they were less active against *M. marinum*. Nevertheless, they serve as useful lead compounds in the search for new drugs and add weight to the theory that a large number of novel compounds remain to be discovered from marine sponges. The Δ^9(11)^ double bond may be beneficial to the design of a compound that is more efficacious and safer than conventional therapy as shown by their inactivity in the cytoxicity assay. It is worth mentioning that the endoperoxide moiety may play a role in the efficacy of the drug at which its potency threshold is above its level of cytoxicity as an antitrypanosomal drug. Such types of compounds are needed in the development of drugs with less toxic side effects.

## Supplementary Files

Supplementary File 1 Supplementary Information (PDF, 3204 KB)
