# Supplementary material for: Isolation and Identification of Antitrypanosomal and Antimycobacterial Active Steroids from the Sponge Haliclona simulans
Source: Mar Drugs. 2014 May 16;12(5):2937–52. doi: 10.3390/md12052937 (PMC4052325; doi:10.3390/md12052937)
Supplement: Supplementary File 1 — Supplementary Information (PDF, 3204 KB) [file marinedrugs-12-02937-s001.pdf]

Supplementary Information

Figure S1. ^{1}H NMR spectrum of 24-vinyl-cholest-9-ene-3β, 24-diol (sterol **2**) (400 MHz).

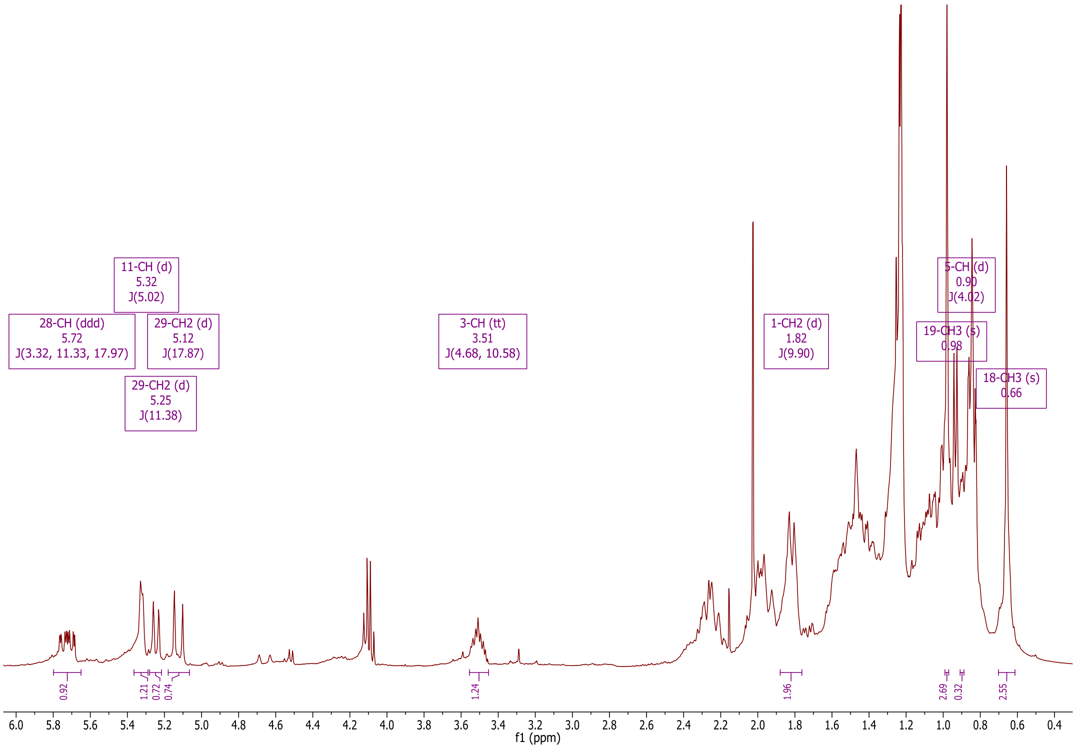

Figure S2. ^{1}H-^{1}H COSY of sterol **2** (400 MHz).

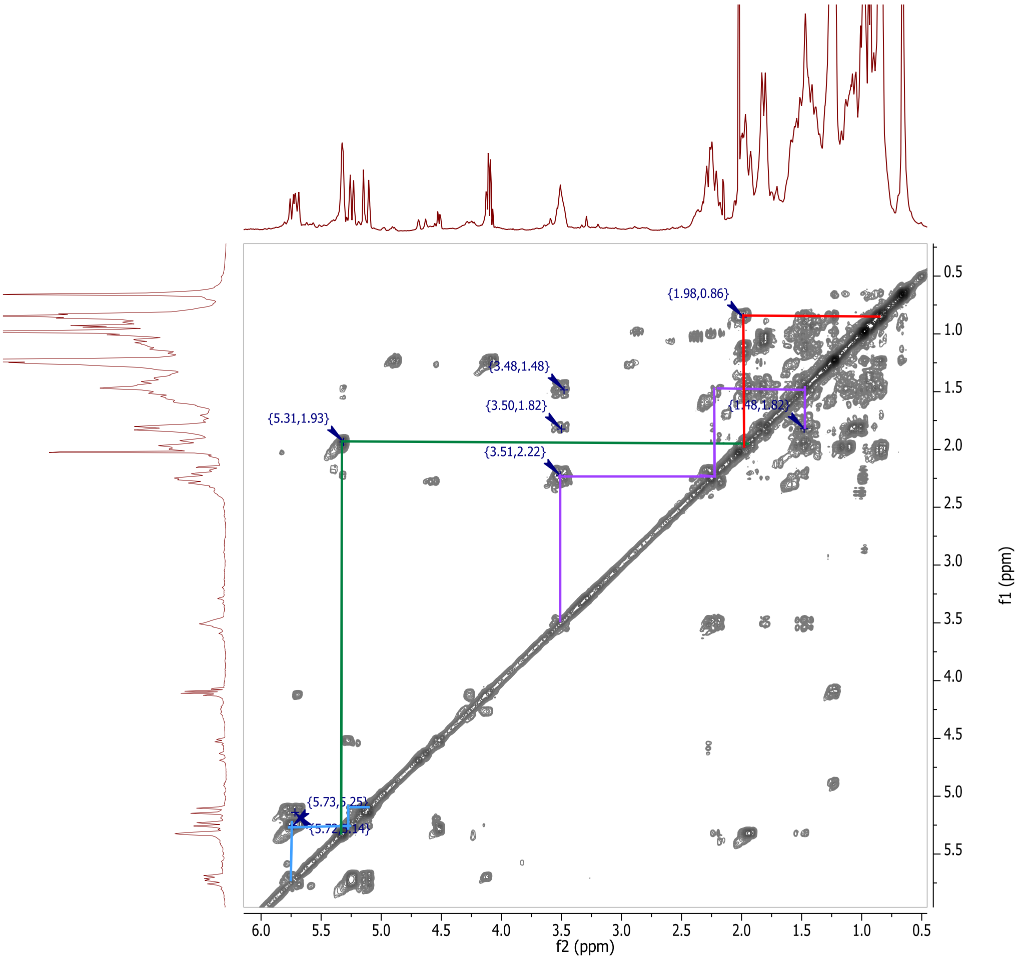

Figure S3. ^{13}C NMR spectrum of sterol **2** (100 MHz).

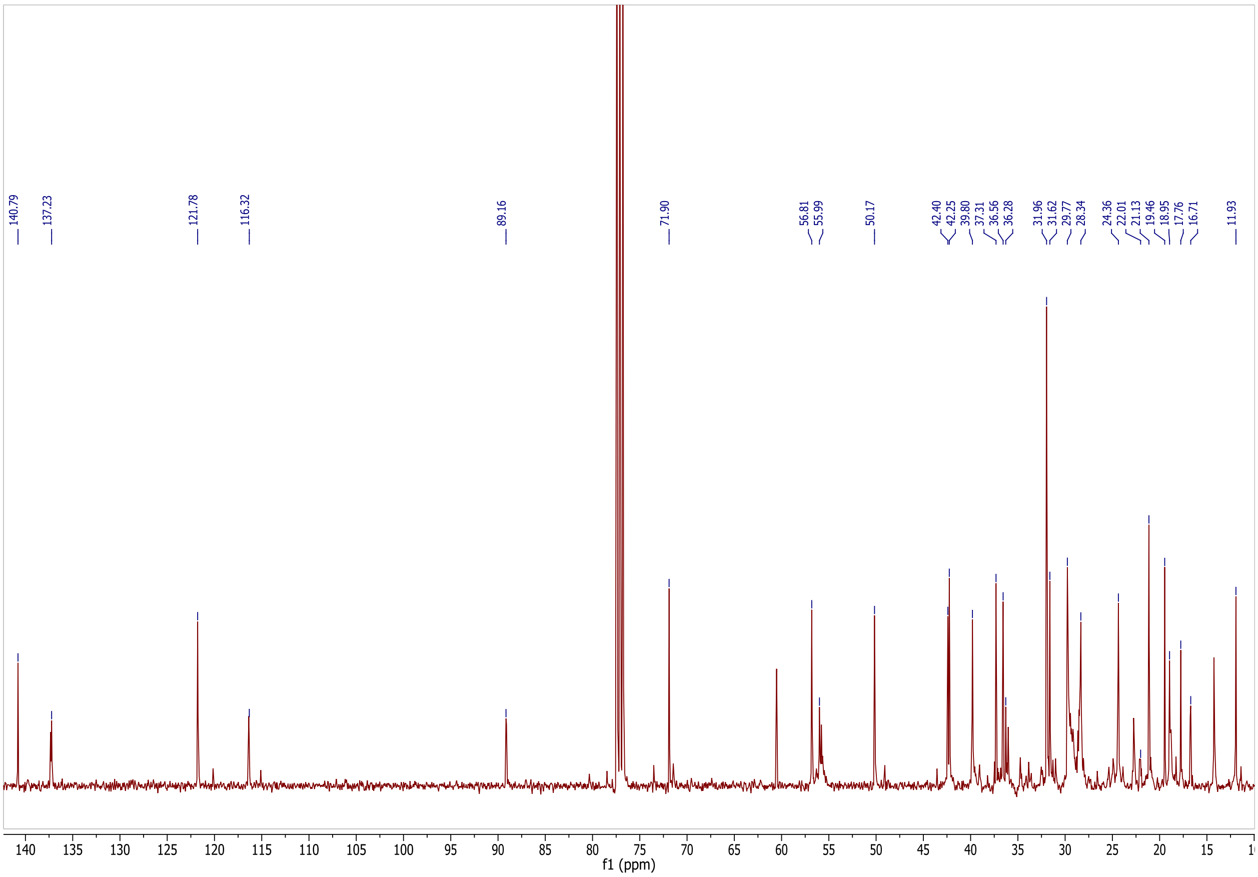

Figure S4. DEPT135 (top) and ^{13}C spectrum (bottom) of sterol **2** (100 MHz).

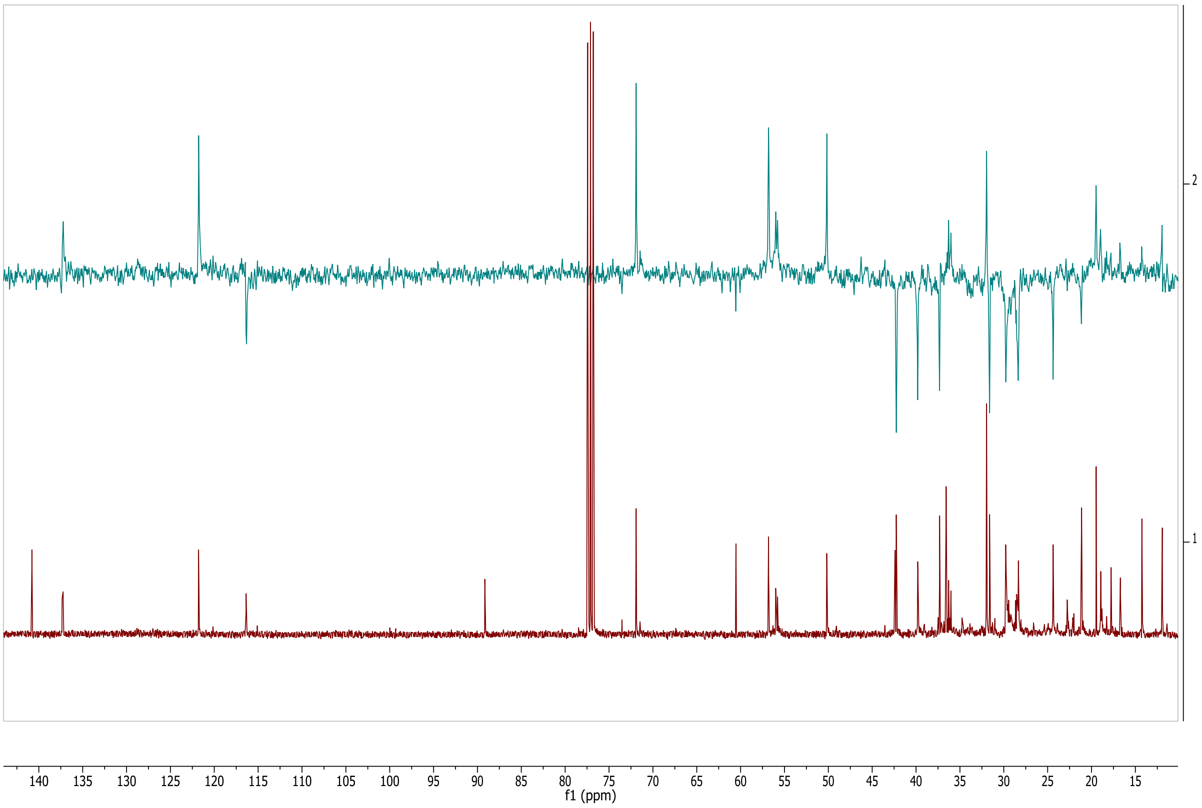

Figure S5. HMQC spectrum of sterol **2** (400 MHz, 100 MHz).

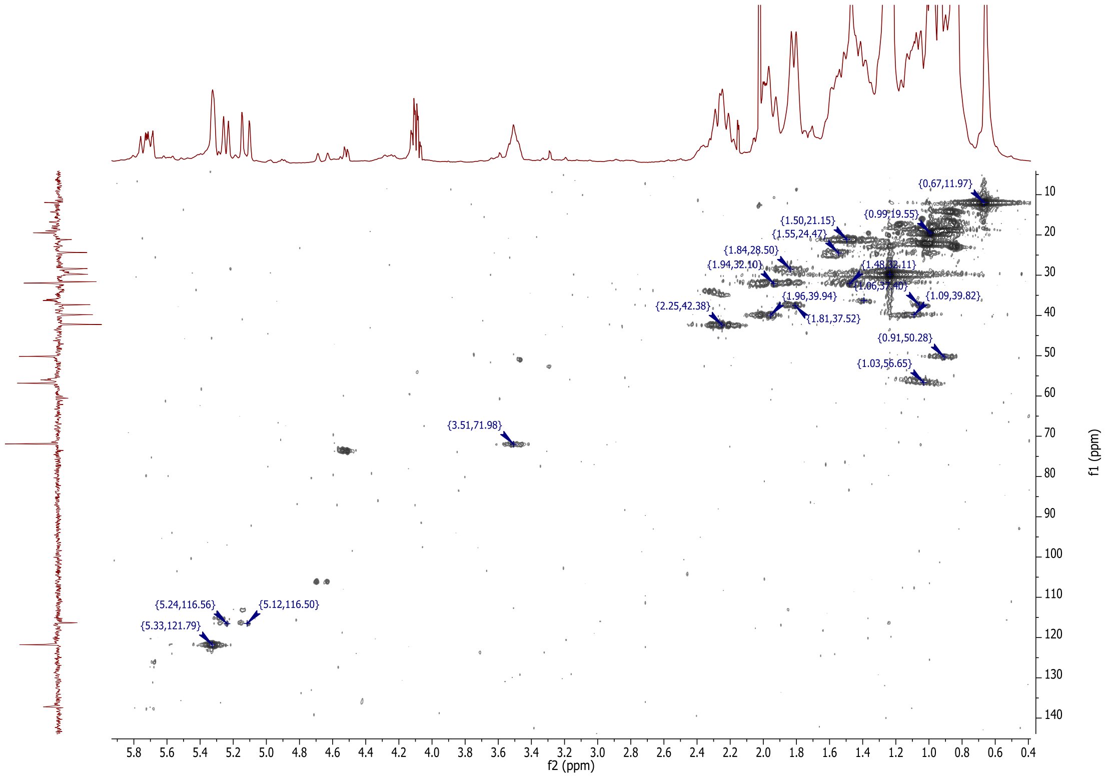

Figure S6. HMBC spectrum of sterol **2** (400 MHz, 100 MHz).

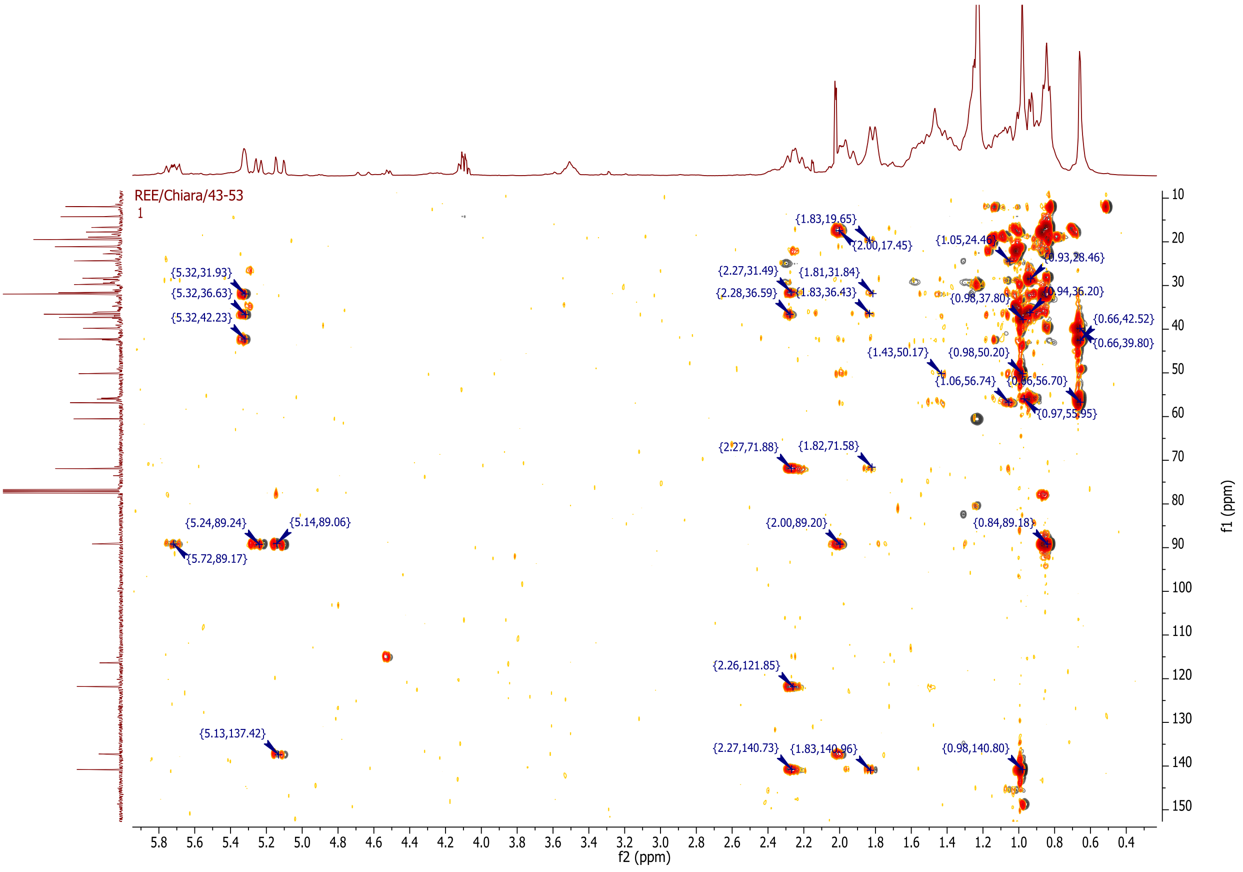

Figure S7. Expansion of the HMBC of sterol **2** highlighting the correlations between H-11 and C-10 and -13, showing the location of the double bond in the steroid nucleus.

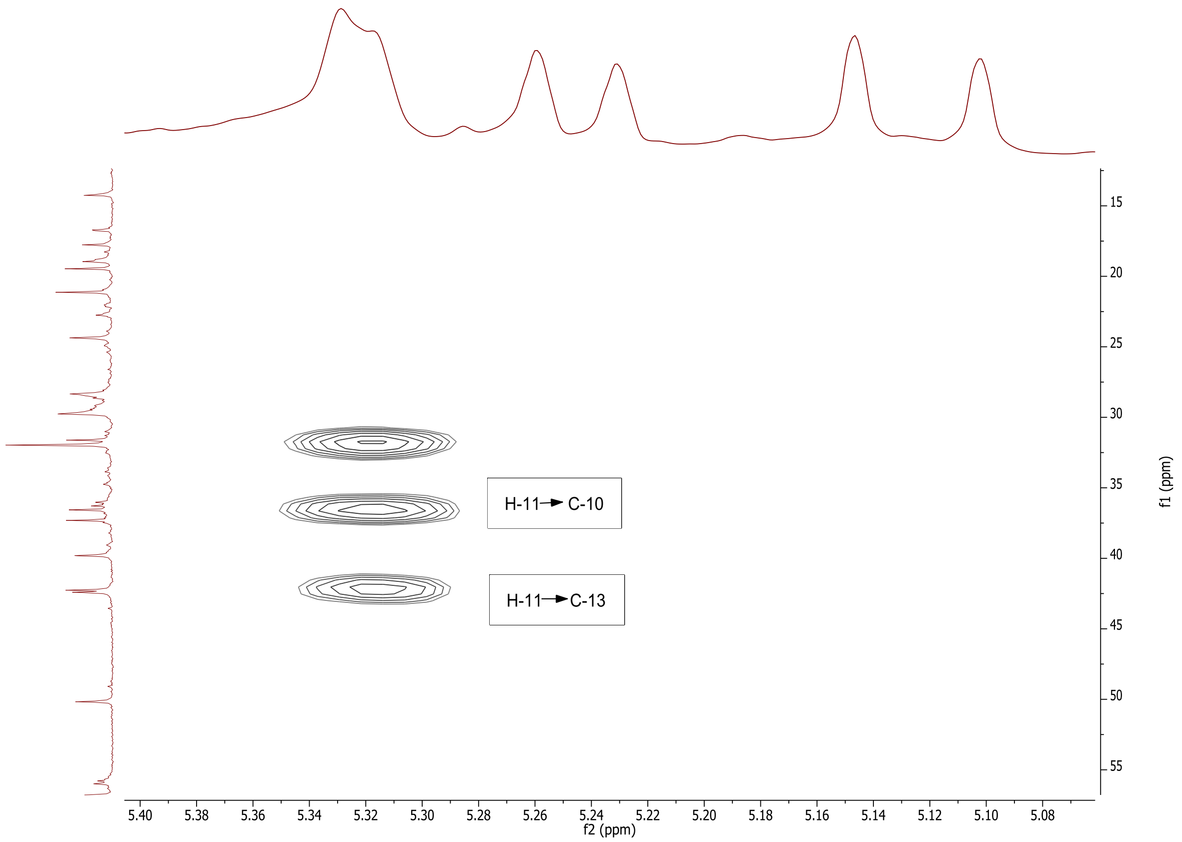

Figure S8. Substructure of sterol **2**. The black arrows indicate the coupling observed between the hydrogens and carbons in the HMBC whereas the light blue and red arrows indicate the coupling observed between neighbouring hydrogens in the COSY.

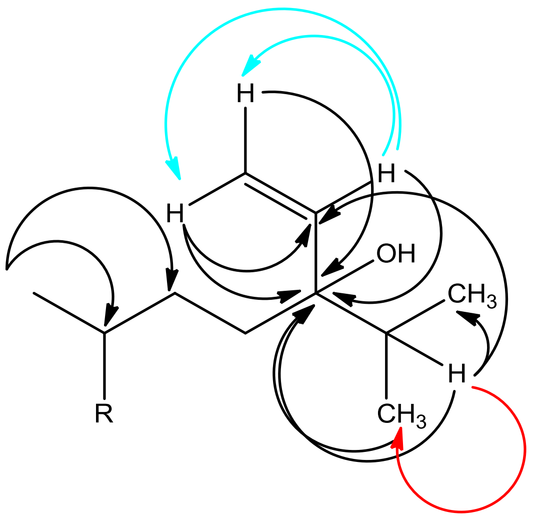

Figure S9. Substructure of sterol **2**. The black arrows indicate the carbons seen by the hydrogens in the HMBC. The purple and green arrows show the ^1H-^1H coupling as seen in the COSY and are coloured by spin system.

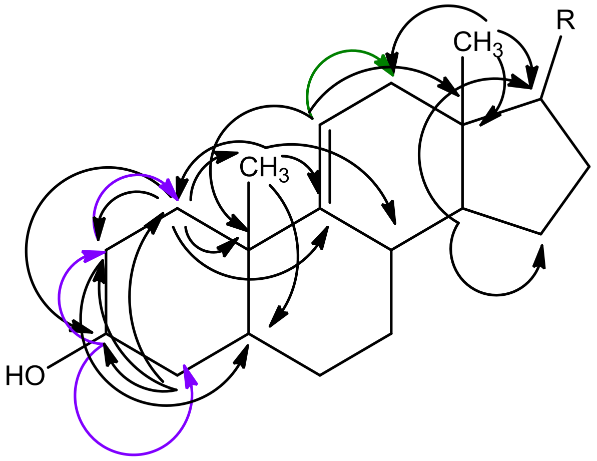

Figure S10. 24-Vinyl-cholest-9-ene-3β, 24-diol (sterol **2**).

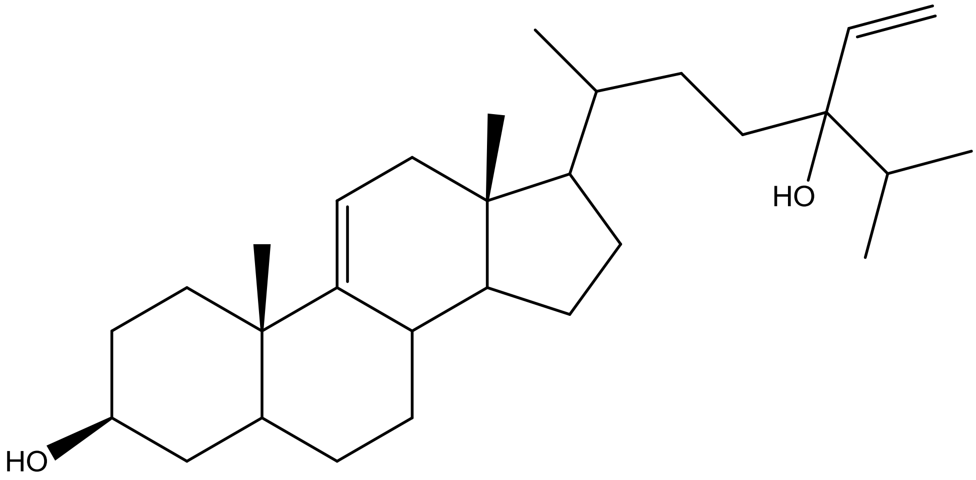

Figure S11. Extracted ion chromatogram (top) and mass spectrum (bottom) of sterol **2** showing the *m/z* of 429.3731 $[M + H]^+$ and a predicted formula of $C_{29}H_{49}O_2$ matching that of the predicted structure.

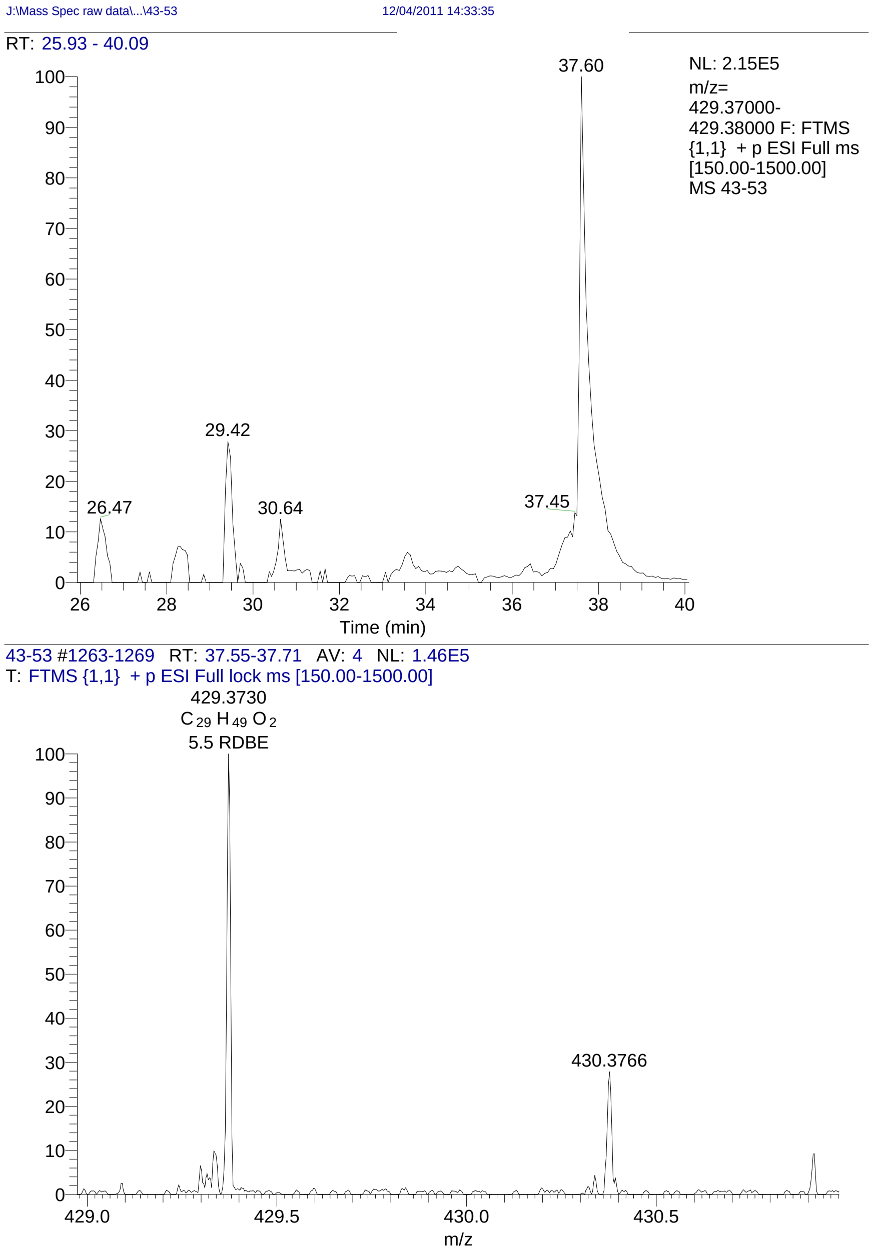

Figure S12. ^{1}H NMR spectrum of 20-methyl-pregn-6-en-3β-ol, 5α,8α-epidioxy (sterol **3**) (400 MHz).

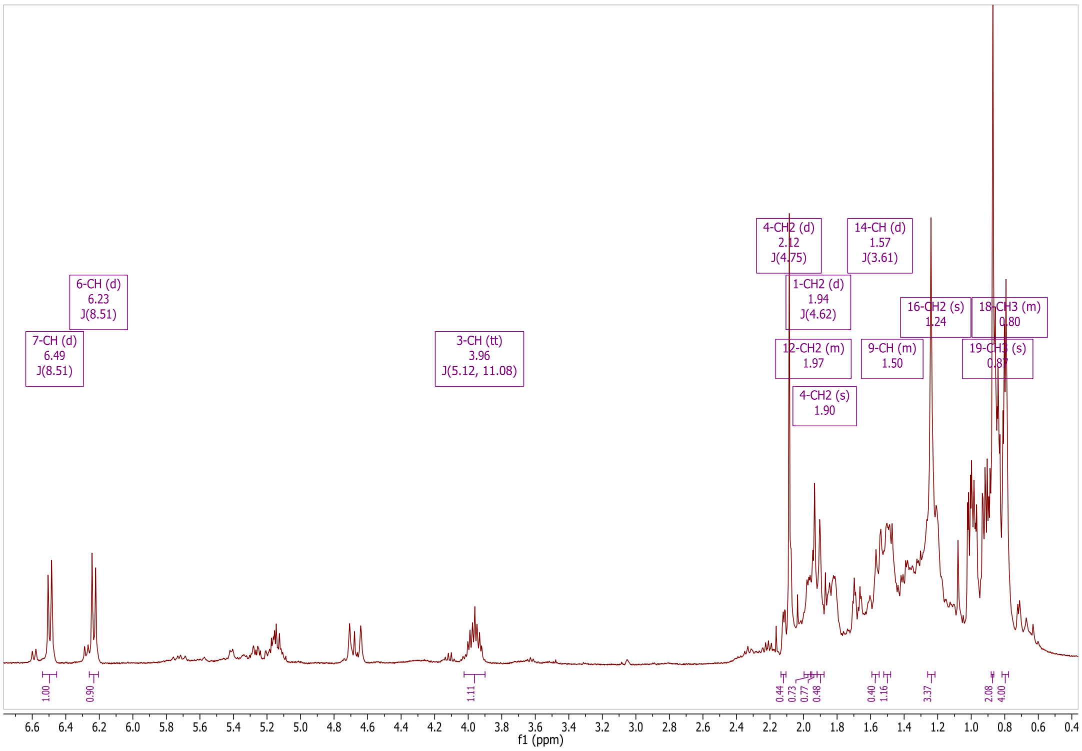

Figure S13. ^{1}H-^{1}H COSY of sterol **3** (400 MHz).

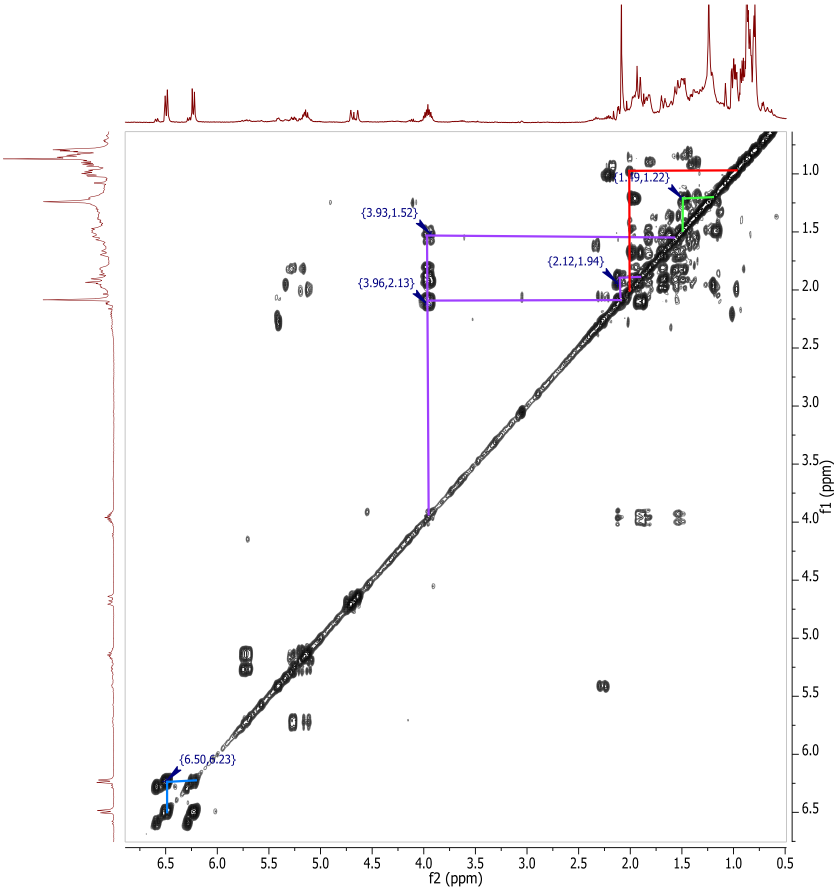

Figure S14. ^{13}C NMR spectrum of sterol **3** (100 MHz).

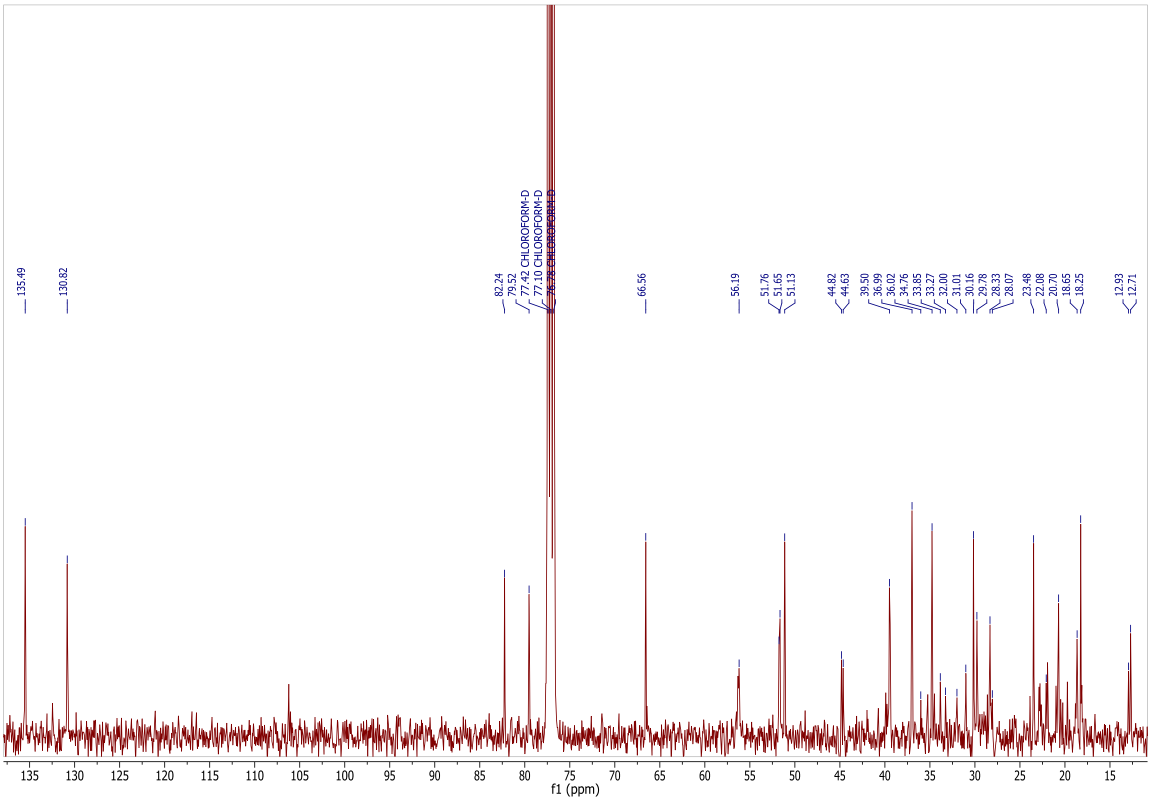

Figure S15. DEPT135 (top) and ^{13}C spectrum (bottom) of sterol **3** (100 MHz).

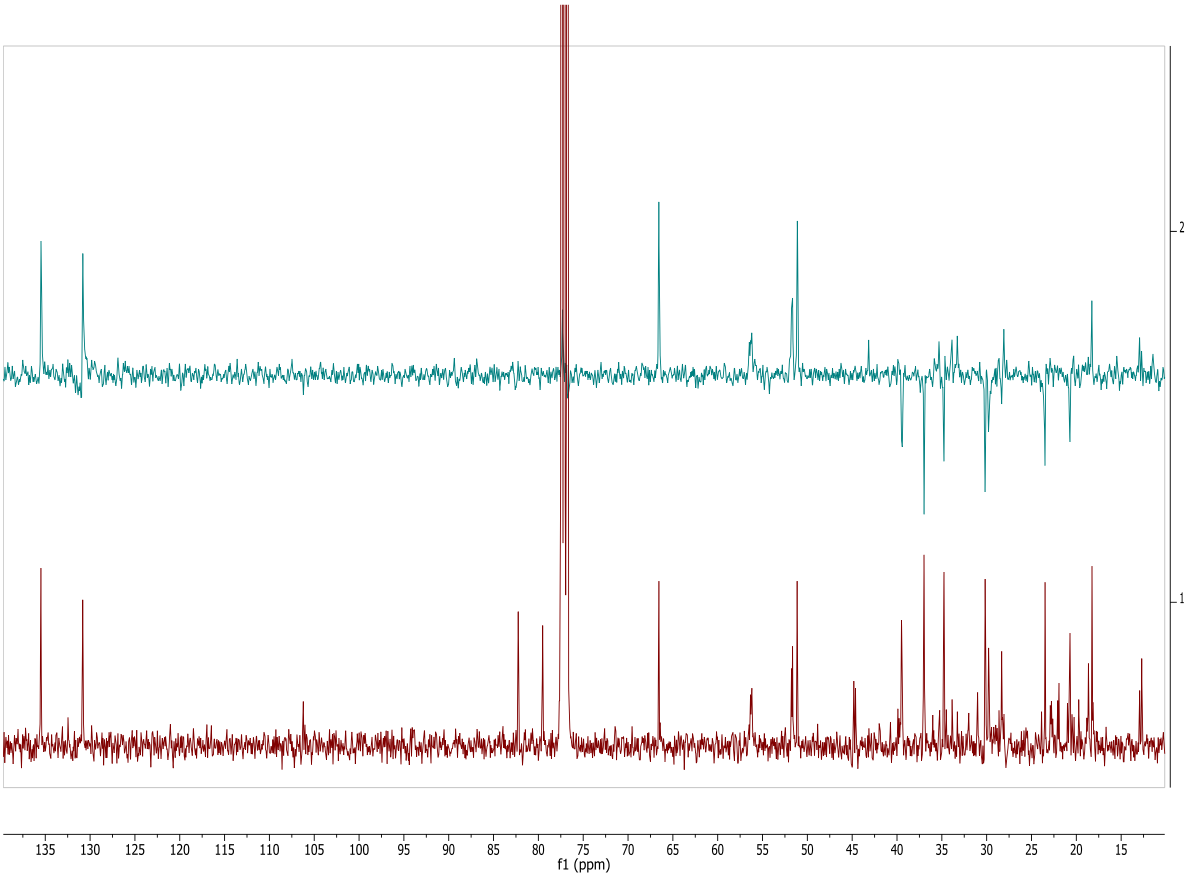

Figure S16. HMQC spectrum of sterol **3** (400 MHz, 100 MHz).

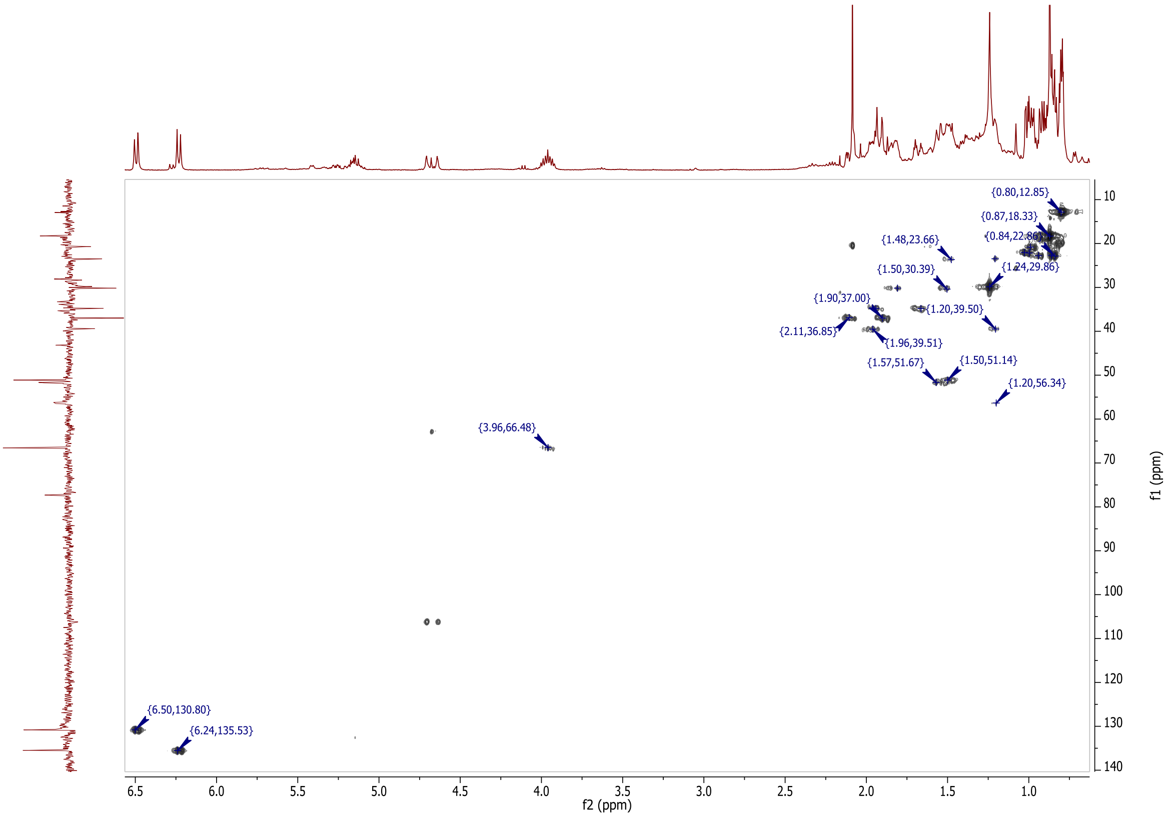

Figure S17. HMBC spectrum of sterol **3** (400 MHz, 100 MHz).

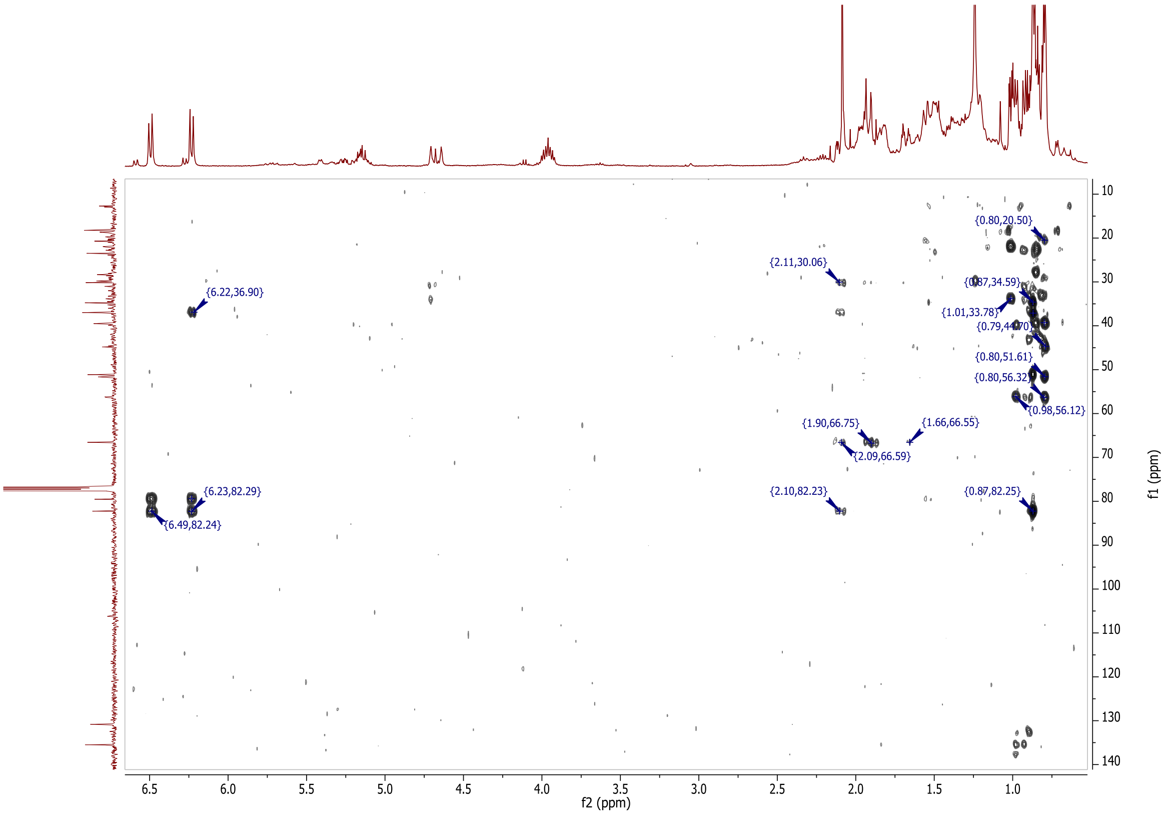

Figure S18. *J*-resolved spectrum of sterol **3**.

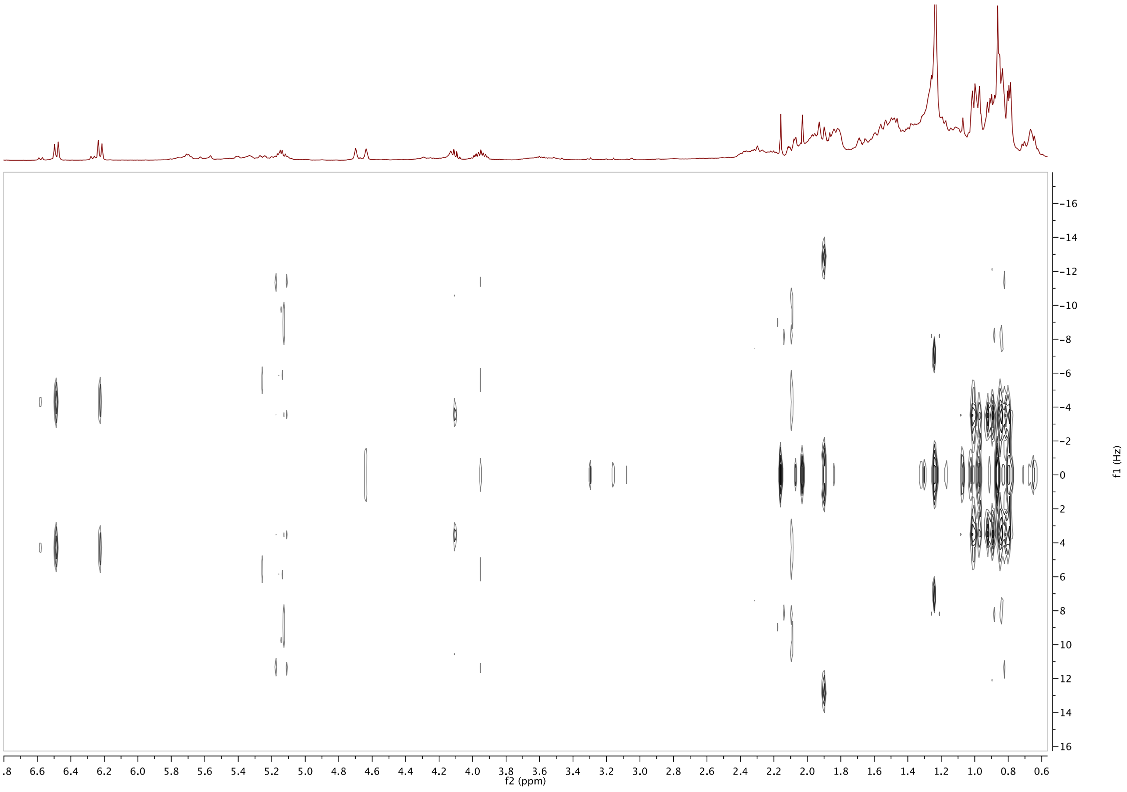

Figure S19. Expansion of the *J*-resolved spectrum showing the splitting of H-21 (δ_H 0.98).

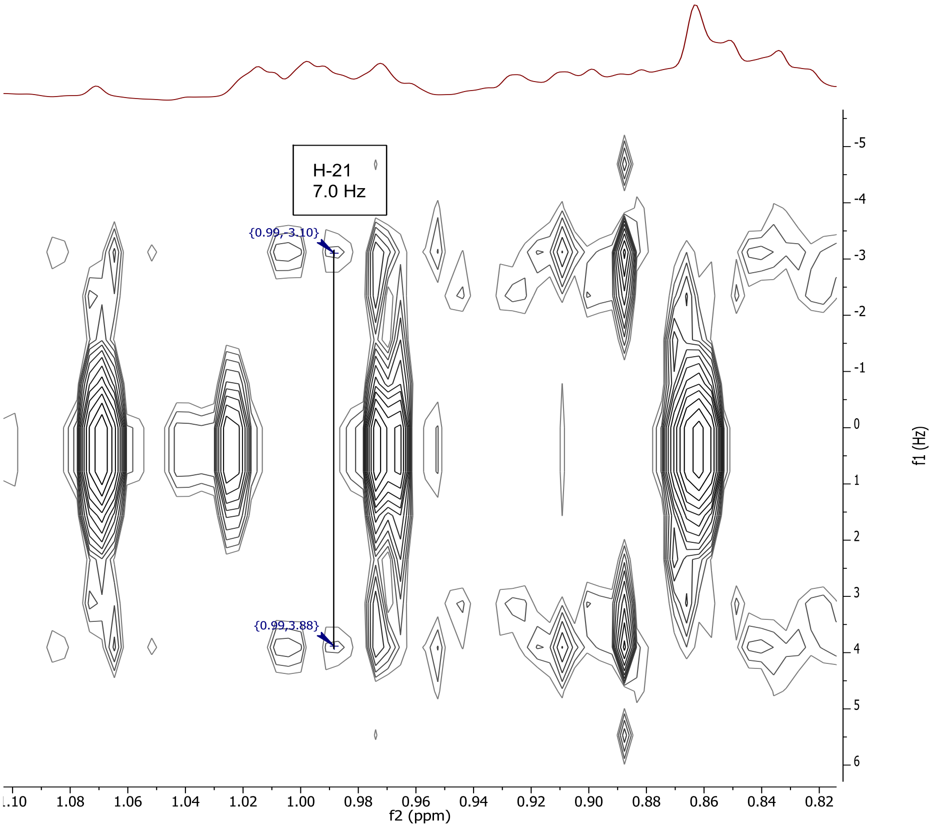

Figure S20. HMBC (black arrows) and ^1H-^1H COSY (coloured arrows) correlations of the proposed structure of sterol **3**.

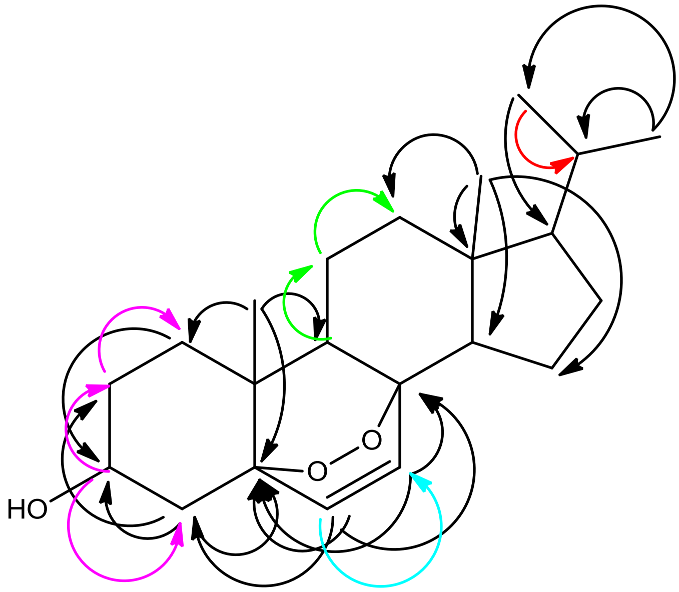

Figure S21. 20-methyl-pregn-6-en-3β-ol, 5α,8α-epidioxy (sterol **3**).

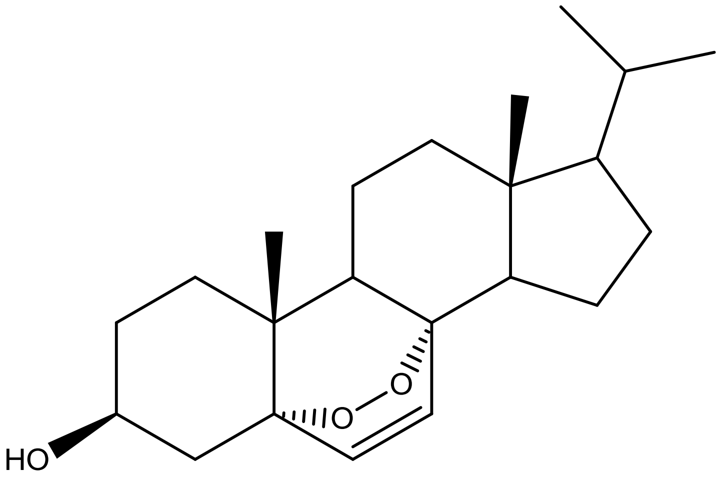

Figure S22. Extracted ion chromatogram of sterol **3** showing *m/z* 347.2580 $[M + H]^+$ and a predicted formula of $C_{22}H_{35}O_3$ matching that of the predicted structure.

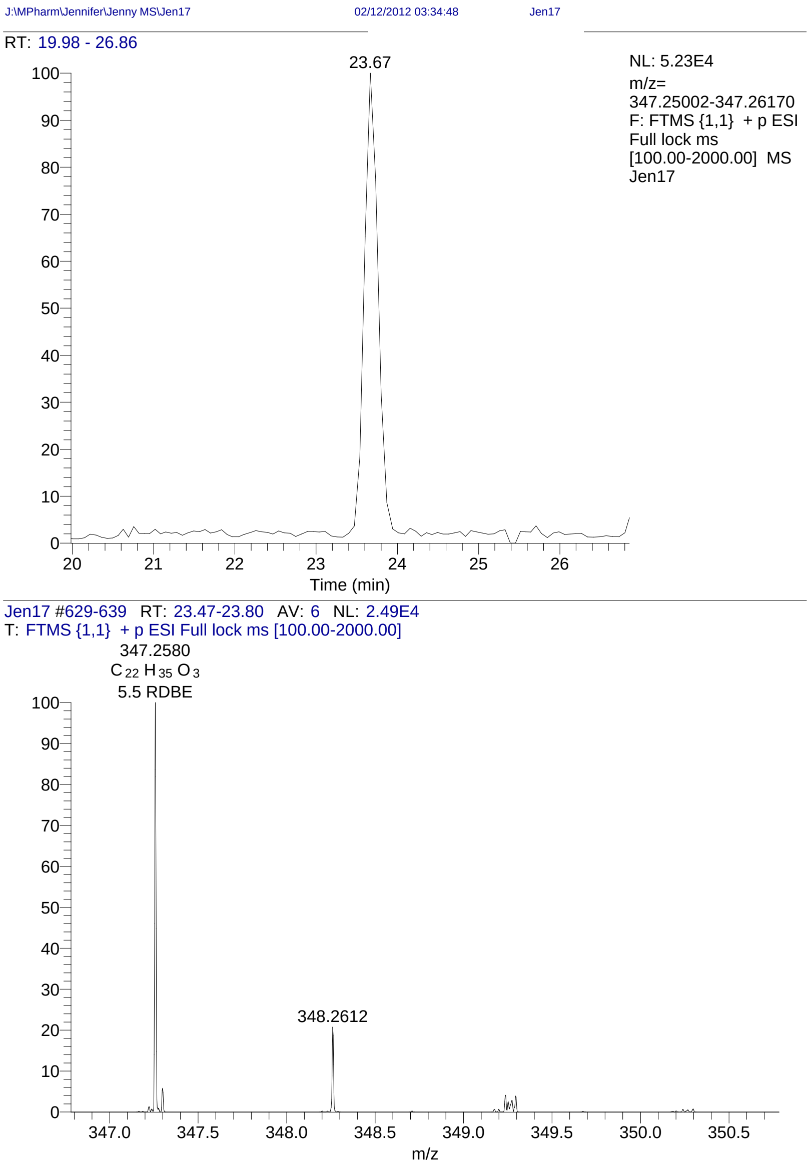

Figure S23. Cytotoxicity of *H. simulans* sterols on HS27 cells.

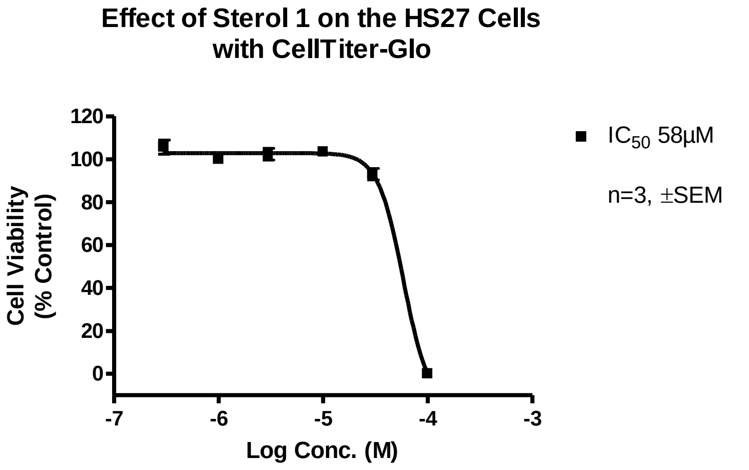

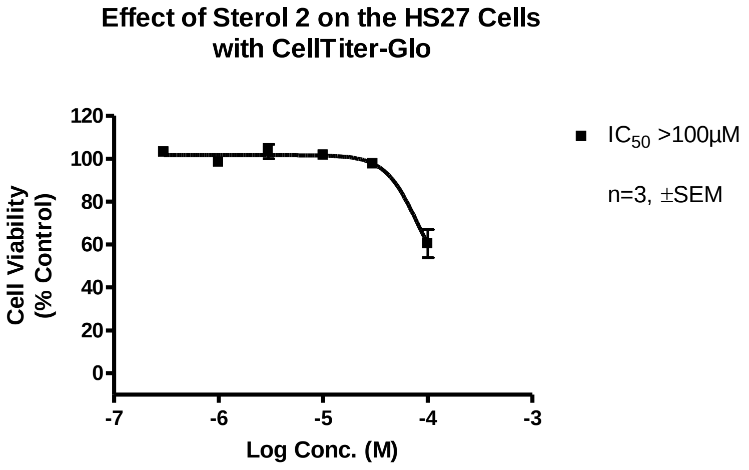

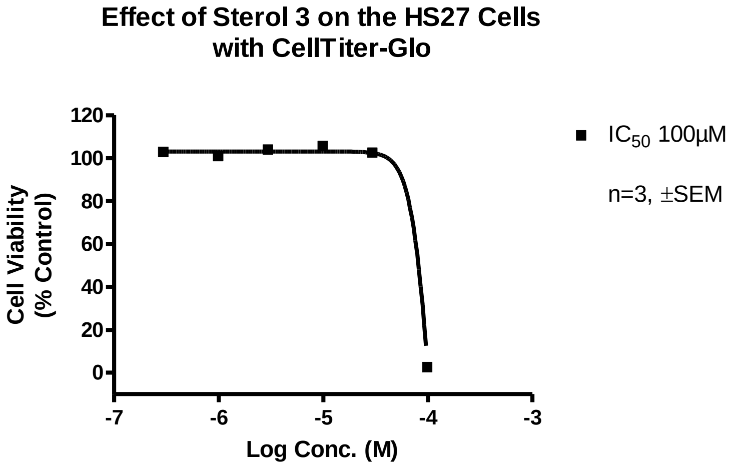

© 2014 by the authors; licensee MDPI, Basel, Switzerland. This article is an open access article distributed under the terms and conditions of the Creative Commons Attribution license (http://creativecommons.org/licenses/by/3.0/).
